# Supplementary material for: Association between systemic immune-inflammation index and chronic kidney disease: A population-based study
Source: PLoS One. 2024 Feb 8;19(2):e0292646. doi: 10.1371/journal.pone.0292646 (PMC10852278; doi:10.1371/journal.pone.0292646)
Supplement: S1 Table — Continuous variables were expressed as mean ± SD, and P-values were calculated by the weighted linear regression model. Categorical variables are shown as percentages: p-values were calculated by weighted chi-square test. BMI, body mass index; SII, systemic immune-inflammation index; CKD: chronic kidney disease. (DOCX) [file pone.0292646.s001.docx]

**Supplementary Table 1 Weighted characteristics of the study population categorized by 5 stages of CKD.**

|  | **Overall** | **CKD** | | | | | | ***p*-Value** |
| --- | --- | --- | --- | --- | --- | --- | --- | --- |
|  |  | **G1** | **G2** | **G3a** | **G3b** | **G4** | **G5** |  |
|  | N=40014 | N=23739  (59.33%) | N=12596  (31.48%) | N=2342  (5.85%) | N=925  (2.31%) | N=290  (0.72%) | N=122  (0.30%) |  |
| Age (Mean ±SD, years) | 49.70 ± 18.02 | 40.55 ± 14.02 | 60.34 ± 14.50 | 71.97 ± 10.12 | 74.68 ± 8.38 | 72.65 ± 11.16 | 61.75 ± 14.02 | <0.001 |
| Gender (%) |  |  |  |  |  |  |  | <0.001 |
| Male | 19412 (48.51%) | 10959 (46.16%) | 6691 (53.12%) | 1154 (49.27%) | 416 (44.97%) | 120 (41.38%) | 72 (59.02%) |  |
| Female | 20602 (51.49%) | 12780 (53.84%) | 5905 (46.88%) | 1188 (50.73%) | 509 (55.03%) | 170 (58.62%) | 50 (40.98%) |  |
| Race (%) |  |  |  |  |  |  |  | <0.001 |
| Mexican American | 6567 (16.41%) | 4881 (20.56%) | 1375 (10.92%) | 183 (7.81%) | 75 (8.11%) | 32 (11.03%) | 21 (17.21%) |  |
| Non-Hispanic White | 17464 (43.64%) | 8382 (35.31%) | 6852 (54.40%) | 1471 (62.81%) | 580 (62.70%) | 151 (52.07%) | 28 (22.95%) |  |
| Non-Hispanic Black | 8215 (20.53%) | 5183 (21.83%) | 2307 (18.32%) | 420 (17.93%) | 178 (19.24%) | 72 (24.83%) | 55 (45.08%) |  |
| Other Hispanic | 3568 (8.92%) | 2384 (10.04%) | 982 (7.80%) | 130 (5.55%) | 49 (5.30%) | 16 (5.52%) | 7 (5.74%) |  |
| Other Race | 4200 (10.50%) | 2909 (12.25%) | 1080 (8.57%) | 138 (5.89%) | 43 (4.65%) | 19 (6.55%) | 11 (9.02%) |  |
| Education (%） |  |  |  |  |  |  |  | <0.001 |
| Less than high school | 10187 (25.49%) | 5994 (25.27%) | 3019 (24.01%) | 689 (29.51%) | 327 (35.43%) | 115 (39.93%) | 43 (35.54%) |  |
| High school | 9237 (23.11%) | 5399 (22.76%) | 2899 (23.05%) | 608 (26.04%) | 236 (25.57%) | 70 (24.31%) | 25 (20.66%) |  |
| More than high school | 20543 (51.40%) | 12331 (51.98%) | 6658 (52.94%) | 1038 (44.45%) | 360 (39.00%) | 103 (35.76%) | 53 (43.80%) |  |
| Marital status (%) |  |  |  |  |  |  |  | <0.001 |
| Never married | 7054 (17.64%) | 5728 (24.14%) | 1120 (8.90%) | 114 (4.87%) | 49 (5.30%) | 17 (5.86%) | 26 (21.31%) |  |
| Maried/Living with  partner | 24034 (60.10%) | 14357 (60.51%) | 7792 (61.89%) | 1265 (54.08%) | 429 (46.43%) | 133 (45.86%) | 58 (47.54%) |  |
| Widowed/Divorced  /Separated | 8904 (22.26%) | 3641 (15.35%) | 3679 (29.22%) | 960 (41.04%) | 446 (48.27%) | 140 (48.28%) | 38 (31.15%) |  |
| Poverty ratio (%) |  |  |  |  |  |  |  | <0.001 |
| 0-1.5 | 25358 (69.23%) | 15396 (70.80%) | 7454 (64.59%) | 1550 (71.89%) | 667 (80.75%) | 201 (80.08%) | 90 (80.36%) |  |
| 1.5-3.5 | 87 (0.24%) | 44 (0.20%) | 43 (0.37%) | 0 (0.00%) | 0 (0.00%) | 0 (0.00%) | 0 (0.00%) |  |
| >3.5 | 11186 (30.54%) | 6305 (29.00%) | 4044 (35.04%) | 606 (28.11%) | 159 (19.25%) | 50 (19.92%) | 22 (19.64%) |  |
| BMI (kg/m², %) |  |  |  |  |  |  |  | <0.001 |
| 0-25 | 11456 (29.03%) | 7418 (31.59%) | 3192 (25.70%) | 542 (23.74%) | 202 (22.82%) | 62 (22.46%) | 40 (34.78%) |  |
| 25-30 | 13135 (33.28%) | 7366 (31.36%) | 4524 (36.42%) | 819 (35.87%) | 308 (34.80%) | 89 (32.25%) | 29 (25.22%) |  |
| >30 | 14875 (37.69%) | 8701 (37.05%) | 4706 (37.88%) | 922 (40.39%) | 375 (42.37%) | 125 (45.29%) | 46 (40.00%) |  |
| Abdominal obesity (%) |  |  |  |  |  |  |  | <0.001 |
| No | 16324 (42.85%) | 10757 (47.07%) | 4619 (38.59%) | 642 (30.27%) | 208 (25.84%) | 53 (21.72%) | 45 (44.55%) |  |
| Yes | 21771 (57.15%) | 12097 (52.93%) | 7351 (61.41%) | 1479 (69.73%) | 597 (74.16%) | 191 (78.28%) | 56 (55.45%) |  |
| Drinking status (%) |  |  |  |  |  |  |  | <0.001 |
| Never | 5166 (14.48%) | 2891 (13.70%) | 1618 (14.23%) | 389 (18.78%) | 174 (21.89%) | 66 (26.72%) | 28 (26.17%) |  |
| Former | 6138 (17.20%) | 2754 (13.05%) | 2355 (20.72%) | 620 (29.94%) | 275 (34.59%) | 94 (38.06%) | 40 (37.38%) |  |
| Mild | 11800 (33.06%) | 6217 (29.46%) | 4447 (39.12%) | 776 (37.47%) | 270 (33.96%) | 60 (24.29%) | 30 (28.04%) |  |
| Moderate | 5429 (15.21%) | 3710 (17.58%) | 1480 (13.02%) | 173 (8.35%) | 42 (5.28%) | 18 (7.29%) | 6 (5.61%) |  |
| Heavy | 7155 (20.05%) | 5529 (26.20%) | 1467 (12.91%) | 113 (5.46%) | 34 (4.28%) | 9 (3.64%) | 3 (2.80%) |  |
| Smoking status (%) |  |  |  |  |  |  |  | <0.001 |
| Never | 21924 (54.83%) | 13681 (57.67%) | 6454 (51.27%) | 1157 (49.44%) | 442 (47.84%) | 125 (43.10%) | 65 (53.28%) |  |
| Former | 9805 (24.52%) | 4288 (18.08%) | 4007 (31.83%) | 937 (40.04%) | 402 (43.51%) | 131 (45.17%) | 40 (32.79%) |  |
| Now | 8257 (20.65%) | 5753 (24.25%) | 2127 (16.90%) | 246 (10.51%) | 80 (8.66%) | 34 (11.72%) | 17 (13.93%) |  |
| Hypertension (%) |  |  |  |  |  |  |  | <0.001 |
| No | 23107 (57.76%) | 16854 (71.01%) | 5562 (44.17%) | 525 (22.42%) | 127 (13.73%) | 30 (10.34%) | 9 (7.38%) |  |
| Yes | 16900 (42.24%) | 6882 (28.99%) | 7030 (55.83%) | 1817 (77.58%) | 798 (86.27%) | 260 (89.66%) | 113 (92.62%) |  |
| Diabetes (%) |  |  |  |  |  |  |  | <0.001 |
| No | 16951 (78.87%) | 13202 (88.10%) | 3511 (67.07%) | 187 (24.83%) | 35 (10.39%) | 10 (7.81%) | 6 (11.32%) |  |
| Yes | 4540 (21.13%) | 1783 (11.90%) | 1724 (32.93%) | 566 (75.17%) | 302 (89.61%) | 118 (92.19%) | 47 (88.68%) |  |
| SII (Mean±SD,1,000cells/µl) | 549.98 ± 374.61 | 539.12 ± 323.83 | 546.74 ± 353.33 | 605.61 ± 698.69 | 657.62 ± 476.48 | 683.07 ± 417.67 | 797.44 ± 983.01 | <0.001 |

Continuous variables were expressed as mean ± SD, and P-values were calculated by the weighted linear regression model. Categorical variables are shown as percentages: p-values were calculated by weighted chi-square test. BMI, body mass index; SII, systemic immune-inflammation index; CKD: chronic kidney disease.
