# Supplementary material for: Association between systemic immune-inflammation index and chronic kidney disease: A population-based study
Source: PLoS One. 2024 Feb 8;19(2):e0292646. doi: 10.1371/journal.pone.0292646 (PMC10852278; doi:10.1371/journal.pone.0292646)
Supplement: S2 Table — Model 1: no covariates were adjusted. Model 2: age, gender, and race were adjusted. Model 3: age, gender, race, marital status, education level, income poverty ratio, BMI, abdominal obesity, drinking status, smoking status, hypertension, and diabetes were adjusted. (DOCX) [file pone.0292646.s002.docx]

**Supplementary Table 2 Association between SII and 5 stages of CKD.**

|  | **Crude**  **Model** | **Partially**  **Adjusted** **Model** | **Fully**  **Adjusted** **Model** |
| --- | --- | --- | --- |
|  | **(Model 1)** | **(Model** **2)** | **(Model3)** |
|  | **OR (95% CI) *p*-Value** | **OR (95% CI) *p*-Value** | **OR (95% CI) *p*-Value** |
| SⅡ/100 | 0.01 (0.01, 0.02) <0.0001 | 0.01 (0.01, 0.01) <0.0001 | 0.01 (0.01, 0.01) <0.0001 |
| SII/100 quartiles |  |  |  |
| Quartile 1  (0.02- 3.36) | 0 | 0 | 0 |
| Quartile 2  (3.36 - 4.73) | -0.01 (-0.03, 0.01) 0.3617 | 0.00 (-0.02, 0.02) 0.8836 | -0.00 (-0.03, 0.02) 0.8509 |
| Quartile 3  (4.73 - 6.69) | 0.02 (0.00, 0.05) 0.0428 | 0.03 (0.01, 0.05) 0.0015 | 0.02 (-0.00, 0.05) 0.0750 |
| Quartile 4  (6.69 - 283.97) | 0.10 (0.08, 0.13) <0.0001 | 0.08 (0.06, 0.10) <0.0001 | 0.05 (0.03, 0.08) <0.0001 |
| *p* for trend | 0.02 (0.02, 0.02) <0.0001 | 0.01 (0.01, 0.02) <0.0001 | 0.01 (0.01, 0.01) <0.0001 |

Model 1: no covariates were adjusted.

Model 2: age, gender, and race were adjusted.

Model 3: age, gender, race, marital status, education level, income poverty ratio, BMI, abdominal obesity, drinking status, smoking status, hypertension, and diabetes were adjusted.
